# Stimuli-Responsive Drug Release from Smart Polymers

**DOI:** 10.3390/jfb10030034

**Published:** 2019-07-31

**Authors:** Carlos M. Wells, Michael Harris, Landon Choi, Vishnu Priya Murali, Fernanda Delbuque Guerra, J. Amber Jennings

**Affiliations:** Department of Biomedical Engineering, The University of Memphis, Memphis, TN 38152, USA

**Keywords:** stimuli-responsiveness, drug release, drug delivery, thermo-responsive materials, enzyme-responsive materials, pH-responsive materials, shape-memory materials

## Abstract

Over the past 10 years, stimuli-responsive polymeric biomaterials have emerged as effective systems for the delivery of therapeutics. Persistent with ongoing efforts to minimize adverse effects, stimuli-responsive biomaterials are designed to release in response to either chemical, physical, or biological triggers. The stimuli-responsiveness of smart biomaterials may improve spatiotemporal specificity of release. The material design may be used to tailor smart polymers to release a drug when particular stimuli are present. Smart biomaterials may use internal or external stimuli as triggering mechanisms. Internal stimuli-responsive smart biomaterials include those that respond to specific enzymes or changes in microenvironment pH; external stimuli can consist of electromagnetic, light, or acoustic energy; with some smart biomaterials responding to multiple stimuli. This review looks at current and evolving stimuli-responsive polymeric biomaterials in their proposed applications.

## 1. Introduction

Researchers throughout different disciplines continue to explore improved and safer ways to locally deliver drugs to specific sites of action, attempting to increase specificity and efficacy. Over the years, numerous investigations on biomaterials have seen successes in the development of controlled delivery systems [1,2]. These achievements have been the results of interdisciplinary contributions across chemistry, biology, physics, and pharmacology, and with inputs from clinicians. Biomaterials have evolved to cover vast libraries, with diverse chemical structures, varied morphologies, and numerous physiological functions. Synthetic polymers, biomacromolecules, nano-/micro-particles, biocompatibility, biodegradability, specific targeting, cargo protection, and bulk materials are some of the developments that have improved or enhanced biomaterials, their applications, or the combination [3]. Drug carriers and their subsequent on-demand release have been the benefactors from flexible designs that evolved to address the perplexing and diverse physiological environment. 

Smart biomaterials are the next evolutionary step in optimizing patient-centric care while potentially providing treatment options previously not available. Smart biomaterials possess tailor-designed stimuli-responsiveness that attempts to address many issues where current drug delivery systems are lacking [4]. The incorporation of different internal stimuli from the physiological microenvironment, such as pH, redox, temperature, enzyme, and mechanical force, or some readily available and relatively easily-controlled external stimuli, e.g., light, ultrasound, electric, and magnetic, has led to highly controlled smart systems [3]. The spatiotemporal control that many smart systems possess increases efficacy, and minimizes potential side effects and off-target toxicity. 

The emphasis of this mini-review is to present and analyze progress in the development of polymeric smart drug delivery biomaterials within the past decade. We will focus on three primary categories for stimuli-responsive polymeric systems, chemical, physical, and biological, with systems containing multiple or overlapping stimuli-responsiveness discussed separately. Within each group, this mini-review will discuss pH-, acoustic-, photo-, magnetic-, electric-, and enzyme-responsiveness as distinct subcategories; a summary of the reviewed studies in tabulated form is present at the conclusion (Table 1). We will survey representative samples for each of the subcategory stimuli types with appropriate advantages, disadvantages, challenges, and future directions. 

## 2. Chemical Stimuli-Responsive Systems

### pH-Responsiveness

Smart biomaterials can be designed to respond to alterations in environmental parameters, promoting the delivery of therapeutics locally when the environmental pH is either acidic or basic, and allowing for a release in particular organ systems or pathological conditions in which the pH changes. For the delivery of highly toxic chemotherapeutics, pH-controlled release systems can limit systemic concentrations of drugs [2,5,6,7,8,9]. The acidic extracellular environment of solid tumors makes it possible to develop pH-sensitive drug delivery systems that undergo physical/chemical changes or both when exposed to acidic pH. Apart from cancer therapy, smart delivery systems explore other diseases, such as gastric ulcers [10], osteomyelitis [11], and diabetes [12]. Generally, polyelectrolyte polymeric systems contain weak acidic or basic groups in the polymer backbone. Changes in environmental pH trigger the acceptance or release of protons, promoting cleavage of bonds, solubility, and or structure. 

**Figure 1:**
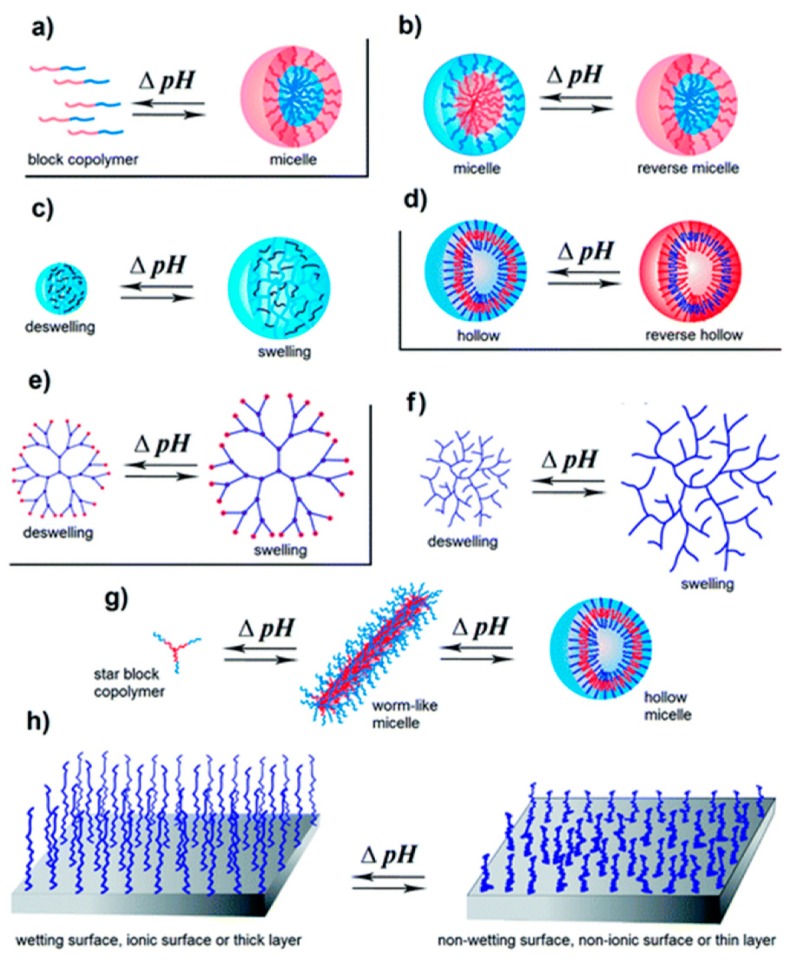
pH-responsive polymers of different architectures: (**a**) unimer–micelle, (**b**) micelle–reverse micelle, (**c**) nanogels or microgels, (**d**) hollow–reverse hollow, (**e**) dendrimer, (**f**) hyper-branched, (**g**) micelle morphology changes (from worm-like to hollow), and (**h**) polymer brushes. Reprinted with permission from *Polymer Chemistry*, 2017, *8*, 144–176. Copyright (2017) The Royal Society of Chemistry.

Materials with pH-responsiveness have been synthesized and studied in a variety of forms, such as hydrogels [6,10,12], nanoparticles [2,8,13,14], beads [15], hollow particles [11], and 3D porous structures [16]. In a recent study, Qu et al. investigated N-carboxyethyl chitosan and dibenzaldehyde-terminated poly(ethylene glycol) (PEG) encapsulated with doxorubicin (DOX) as an injectable hydrogel for hepatocellular carcinoma therapy [6]. The study demonstrated how changes in pH promote chemical and physical modifications to swell the polymeric system, which causes the release of the encapsulated drug. Using a similar concept, Li et al. fabricated fluorescent boronate nanoparticles encapsulated with DOX for intracellular imaging and suppression of a cancer cell line, Michigan Cancer Foundation-7, commonly referred to as MCF-7 [2]. In their study, the system was created based on the formation of a boronic ester link between two different acid-cleavable modified poly(lactic acid)–poly(ethyleneimine) (PLA–PEI) copolymer moieties, boronic acid, and a diol. These biomaterials demonstrated a burst release of DOX after a minimal initial release upon a pH shift from 7.4 to 5.4. Representative polymeric systems and their pH-responsiveness can be seen in Figure 1 [17].

In a different approach, a group investigated the use of sodium bicarbonate inside poly(lactic-co-glycolic acid) (PLGA) hollow microspheres [11]. Instead of acid-cleavable bonds stimulating response, this system relied on a chemical reaction within the delivery system, which yields by-products that stimulate a change in the morphology of the material encapsulating the drug. In acidic conditions, bicarbonate generates CO_2_ and water from the decomposition of the carbonic acid, building the gas pressure to burst the PLGA shell and thus release the encapsulated antibiotic. Similarly to cancerous tissues, inflamed tissues have a lower pH, and therefore, the pH-sensitive release has advantages for infection therapy. The authors reported that in a preclinical model, infections were successfully cured only in the group that used the pH-sensitive material for antibiotic delivery. 

Other research has explored materials that are sensitive to higher pH levels to target organs, such as the lower intestine. In a study reported by Pafiti et al., hydrogels composed of collapsed hollow particles entrapped within a poly(acrylamide) network demonstrated disintegration and drug release at pH 8.0 [18]. This pH-sensitive material was capable of maintaining its initial morphological properties in the stomach (pH < 4.0) and promoted the release of a drug due to the disintegration of the collapsed hollow particles in high pH. 

Polymeric materials with pH-responsiveness have demonstrated tailoring for specific applications. pH-responsive polymers allow physicochemical features, such as the degradation rate, solubility, flexibility, injectability, adhesion, and mechanical strength, to be modulated. Furthermore, the ability to produce polymeric biomaterials in different forms and shapes allows for the administration of these systems in a variety of methods, including intravenous, intraosseous, subcutaneous, ingestion, and transdermal. A disadvantage of pH-responsive systems requires that the environmental pH may vary depending on the severity of disease or proximity to diseased tissue and maintaining structure during the process of delivery may be challenging. pH-sensitive systems may be activated during implantation or administration, making these systems susceptible to off-target delivery. Overcoming these disadvantages concurrent with progress on the existent advantages provides room for future research, development, and improvements in pH-responsive polymers for drug delivery.

## 3. Physical Stimuli-Responsive Systems

### 3.1. Acoustic-Responsiveness

Through the incorporation of microarchitecture and steric hindrance, PEGylated gold-nanoparticles encapsulated within calcium cross-linked alginate microbeads hydrogels have been investigated for their ability to release bone morphogenetic protein-2 conjugated gold nanoparticles through an “on/off” switch [19]. The use of ultrasound (U/S) led to a six-fold increase in the cumulative release of PEGylated gold nanoparticles compared to the use of no stimuli (diffusion), with the demonstration of repeatability of acoustic stimuli-responsiveness [19]. Huebsch et al. designed a similar system for an injectable digital drug release involving the use of ultrasound to trigger the release of the cancer therapeutic mitoxantrone encapsulated in cross-linked self-healing alginate hydrogels [20]. This study found that pulsed stimulation outperformed constant ultrasound. These cross-linked alginate systems implanted into xenograft tumors in mice were more sensitive to short-term, high-dose “bursts” of the chemotherapeutic mitoxantrone than to continuous doses over more extended periods [20]. 

**Figure 2:**
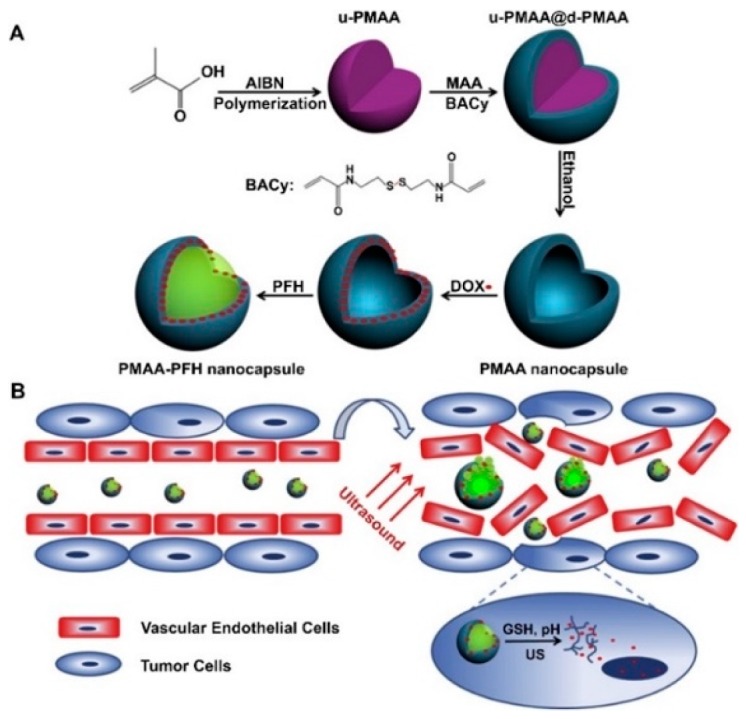
(**A**) Schematic representation of the preparation of doxorubicin loaded poly(methacylate acid)-perfluorohexane (PMAA-PFH) nanocapsules. (**B**) Schematic procedure for imaging-guided ultrasound triggered drug delivery to tumors using biodegradable PMAA-PFH nanocapsules. Reprinted with permission from *Biomaterials*, 2014, *35(6),* 2079–2088. Copyright (2014) Elsevier Ltd.

Several systems make use of capsules or bubbles to enable acoustic responses due to cavitation. Zhou et al. investigated biocompatible chitosan nanobubbles (NBs) suitable for U/S-targeted DOX delivery, showing significant increases in DOX release after ultrasound stimulation for its noninvasive release profile. The in vitro release profile of the DOX-NBs in the U/S group had released a substantial amount of the encapsulated DOX compared to the non-U/S group, and nearly twice as much after 24 h [21]. Yang et al. designed uniform biodegradable nanocapsules to form a three-in-one theranostic nanoplatform. In this system, perfluorohexane was encapsulated by DOX-loaded poly(methacrylic acid) (PMAA) shells with disulfide cross-linkage for ultrasound readings and drug release (Figure 2) [22]. A combination of acidic pH exposure to glutathione and ultrasound stimulation resulted in an increased release [22]. Nguyen et al. produced stable nanodroplets that release simvastatin for degenerative disc disease that can undergo multiple exposures of high-intensity focused ultrasound (HIFU) for possible long-term treatment. Compared to the control exposure (sham), the U/S-treatment doubled the drug release and exhibited a consistent drug release with each U/S-exposure [23]. 

Cao et al. devised phase-changeable materials composed of lipid-base and PLGA nanodroplets that release DOX and perfluoropentane when triggered by low-intensity focused ultrasound (LIFU) to improve anticancer drug delivery [24]. The nanodroplets demonstrated improved inhibition of tumor proliferation over materials not treated with LIFU, leading to enhanced animal survival [24]. Increasing the number of pulses improved the animal survival rate compared to a single burst of LIFU; an increase in vascular permeability of tumors was additionally observed [24]. Salgarella et al. investigated the synthesis and evaluation of five different forms of poly(2-oxazoline) micelles for a possible carrier of a drug delivery system triggered with ultrasound [25]. Micelles were tailored by controlling the ratio of hydrophilic and hydrophobic block copolymers and exhibited a significant release of dexamethasone. Gai et al. developed free-standing biocompatible polylactic acid (PLA) nano- and micro-chamber arrays using two different methods for encapsulation, one-step dip-coating and microcontact printing of air, NaCl, and rhodamine B dye [26]. This work showed that the formation of microchambers can provide long-term encapsulation of small hydrophilic molecules and the release profiles of the microchambers with and without the use of HIFU [26]. With the use of HIFU treatment, the microchambers release around four times the amount of the group without the HIFU treatment [26]. 

The utilization of acoustic-responsive systems provides efficient drug release and controlled elution based upon the composition of the carriers before and after the use of high- or low-frequency ultrasound. Ultrasound as a stimulus for drug delivery may use readily available equipment and is relatively noninvasive. There may be limitations in how deep within tissue the ultrasound can penetrate; with an ongoing need to investigate how to deliver the materials to targeted tissues before stimulation. Exploration of pulsed vs. constant stimulation could enable further tailoring of targeted therapies with new diagnostic qualities. 

### 3.2. Photo-Responsiveness

Light-responsive biomaterials have been an attractive option for the controlled release of drugs and other therapeutic molecules, as they can be induced non-invasively with high spatial and temporal precision [27,28]. Such biomaterials reduce the overall systemic dosage of the drug, thereby reducing the side effects and providing prolonged action at the target site [29,30]. Most commonly used light-responsive agents include spiropyrans (SP) and azobenzenes (Azo) [28,31]. Hydrophobic SP upon ultraviolet (UV) irradiation reversibly changes from its nonionic form to a hydrophilic polar isomer called merocyanine, which reverts to SP upon exposure to visible light [32,33,34]. Azo is another photo-responsive agent, which switches reversibly from its more stable and apolar *trans*-state to a more polar *cis* state upon UV irradiation [28,35]. Reversion induction occurs by more prolonged wavelength exposure or thermal relaxation [35]. 

**Figure 3:**
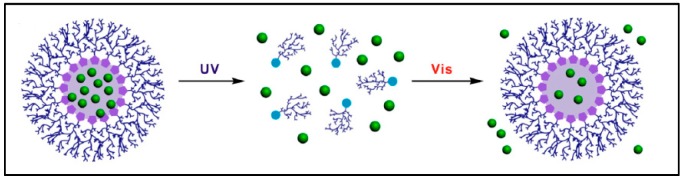
Illustration of model drug (green spheres) release upon 254 nm UV irradiation and re-encapsulation upon 620 nm visible irradiation of spiropyrans-hyperbranched polyglycerol micelles. Reprinted with permission from *Biomacromolecules* **2014**, *15*, 628–634. Copyright (2014) American Chemical Society.

Ultraviolet light has been commonly used to induce drug release in light-responsive biomaterials since these biomaterials mostly respond to shorter wavelengths of light. Kim et al. used SP to synthesize hyperbranched polyglycerol micelles, which could be used to load and release therapeutics in a site-specific and time-controlled manner (Figure 3) [34]. Upon UV exposure, SP photo-isomerized to hydrophilic merocyanine, causing the disassembly of the micelle and stimulating the controlled release of model hydrophobic drugs. Merocyanine reverted to SP upon visible light exposure [34]. 

To develop targeted cancer drug delivery systems, Pearson et al. synthesized light-responsive glycopolymer micelles made of Azo and β-galactose units [36]. The galactose units were intended to target the galectin-3-receptors, overexpressed on melanoma cells. The Azo groups controlled the release of the hydrophobic model drug, attributed to copolymer disassembly. Hardy et al. developed a hydrogel-based “on-demand” micro-needle array transdermal drug delivery system, made from 2-hydroxyethyl methacrylate and ethylene glycol dimethacrylate as well as ibuprofen-loaded 3,5-dimethoxybenzene conjugate [37]. These microneedles containing the conjugate and drug were inserted in the skin and irradiated with UV light, stimulating cleavage of the conjugate and releasing ibuprofen as the microneedle array hydrogel swelled. In vitro, this system remained intact and delivered multiple doses of the drug upon application of an optical trigger [37]. To overcome the multidrug resistance (MDR) responsible for the low effectiveness of chemotherapeutics, Chen et al. developed light-responsive mPEG-PLGA nanoparticles that induced nitric oxide (NO) release when exposed to UV, which reversed the MDR of tumor cells, in addition to breaking open nanoparticle shells to release DOX [38]. 

Primary limitations of UV-stimulated release are poor tissue penetration and its damaging effects on healthy tissues [39]. To avoid them, scientists are shifting the focus towards developing near-infrared (NIR) or visible light-responsive material. Tian et al. developed a diselenide cross-linked polymethacrylic acid system loaded with DOX and indocyanine green [40]. Upon NIR irradiation, indocyanine green released reactive oxygen species, which cleaved the diselenide bond, disrupting the nanogel and releasing DOX. In the absence of NIR, meager amounts of DOX from the nanogels were released, contrasted with the maximum release and toxicity to cancer cells when irradiated with NIR [40]. Photodynamic therapy/photothermal therapy or both provides the basis for many NIR light-responsive systems. Liang et al. developed a hybrid nanoparticle system made up of an NIR light-responsive chromophore (DEACM), incorporated to β-cyclodextrins (β-CD) with cRGD functionalized PEG and coordinated with DOX-loaded AuNRs [41]. On NIR light exposure, Au enhanced the photosolvolysis of DEACM and triggered the release of DOX. The system showed improved anti-cancer effect in vitro as well as in vivo [41]. An “on-demand” drug delivery system was developed based on polymer-nanostructure composite microneedles of polycaprolactone and NIR absorbing LaB_6_@SiO_2_ reported by Chen et al. [42]. Upon NIR exposure, the LaB_6_@SiO_2_ mediated a light to heat transduction, causing the melting of the microneedles, and releasing the model drug, rhodamine 6G dye [42]. Wang et al. developed tetra-ortho-methoxy substituted azobenzene (mAzo), which responded to red light instead of UV light [43,44]. Upon red light exposure, the compound underwent disassembly and a gel-to-sol transition, releasing model proteins. The drug release stopped when the red light was switched to blue light [44]. 

Photo-responsive biomaterials are practical options for the controlled release of therapeutics as they can be induced non-invasively with high precision, thereby increasing their action at the target site and reducing systemic toxicity. Current light sources in use and investigated include UV, visible, NIR, and lasers of various wavelengths. Despite recent progress, many light-responsive polymers have incredibly complicated and technique-sensitive synthesis processes, which limits their ability to be produced in bulk. Though NIR-responsive agents are gaining popularity over UV-responsive agents because of the inadequate tissue-penetrating ability of UV, there are no studies that have investigated NIR-responsive agents in deep tissues. The few studies which have tested these agents in vivo have used only superficial disease models. Also, NIR light-responsive systems have lower efficiency and thereby would require a prolonged exposure time to produce a practical therapeutic effect, which may cause damage to the surrounding healthy tissue due to unwanted excessive heating. The focus is shifting toward biodegradable systems, with improved cytocompatibility and those that naturally degrade in the body once the loaded drug is released. Most of these studies have been carried out only in vitro. To better understand their translational potential, it is essential to continue development and confirm results in vivo.

### 3.3. Magnetic-Responsiveness

Magnetic stimulation is unique in that it can be used to target the drug delivery system, monitor the concentration and distribution of the system, and influence the rate drug release, enabling precise control over the location and rate of drug delivery. These materials typically contain Fe_3_O_4_ superparamagnetic iron oxide nanoparticles (SPIONs), which offer a favorable combination of biocompatibility and magnetic responsiveness. At sufficiently small sizes, typically 100 nm or less, iron oxide nanoparticles exhibit superparamagnetic behavior in which thermal fluctuations randomize the magnetic moment of the particles and eliminate residual magnetization that might trigger particle agglomeration in vivo [45]. Magnetic guidance can be accomplished using a series of permanent magnets placed around the exterior of the subject to create a magnetic field gradient that retains and removes SPIONS from the bloodstream. SPION stimulation with an alternating-current magnetic field (AMF) induces heat generation through Brownian losses, Neel relaxation, or both. This heat can be utilized to increase drug diffusion from the drug delivery system, induce conformational changes or pore formation in polymers surrounding the SPION, or break heat-labile covalent bonds to increase the rate of drug release on demand [45].

**Figure 4:**
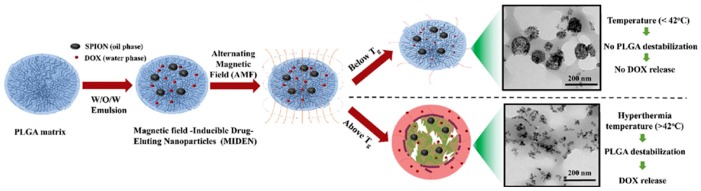
Schematic illustration showing the application of alternating-current magnetic field to induce a phase transition in poly(lactic-co-glycolic acid) nanoparticles and increase the release of a chemotherapeutic. Reprinted with permission from *Biomaterials* 2018, *180*, 240–252. Copyright (2018) Elsevier Ltd.

Clinical efficacy and safety of SPIONS have been demonstrated in vivo, with several clinical trials showing that magnetic stimulation of SPIONS was well-tolerated in vivo and could reliably maintain temperatures between 40 and 45 °C in glioblastoma and prostate tumors [46,47]. SPIONS generate high local temperatures that decay exponentially with distance from the surface [48]; at sufficiently low concentrations, SPION hyperthermia can be used to drive drug release without significantly raising the temperature of the surrounding tissue. Recent studies have effectively measured the local temperature surrounding AMF-stimulated SPIONS and shown temperature increases of up to 50 °C at the surface (<1 nm) without significantly increasing the temperature of the surrounding media [49,50]. These findings suggest that SPION-based hyperthermia can be useful with heat-labile bonds or thermosensitive materials with critical temperatures above the threshold for hyperthermic tissue damage. For example, AMF can be used to induce a phase transition in SPION-loaded PLGA nanoparticles with a glass transition temperature (T_g_) of 42 °C, thereby doubling the amount of DOX released compared to particles with higher T_g_ (Figure 4) [51]. Riedinger et al. used varying lengths of polyethylene glycol to space a thermolabile azo linker at various distances from the surface of the SPIONs, achieving up to threefold increases in DOX elution when stimulated with AMF by tailoring the spacer length [48]. The use of varying spacer lengths in an SPION could theoretically be used to provide further fine-tuning of stimuli-responsiveness or create a different release profile for dual drug systems.

The ability to externally modulate drug release is particularly advantageous as magnetic field generators can be used to increase the drug concentration for acute symptom flares. One particularly appealing application is the delivery of nonsteroidal anti-inflammatory drugs (NSAIDs), as patients would be able to self-treat acute flares of chronic musculoskeletal pain through SPION-mediated drug delivery, thereby reducing the need for opioid pain medications and repeat doctor visits [52,53]. In a murine model of analgesia, magnetic stimulation of ketorolac-loaded SPIONs provided a 50% increase in the duration of clinically assessed pain relief compared to non-stimulated particles, while both provided greater magnitude and duration of pain relief compared to ketorolac alone [52]. Another study by Duan et al. demonstrated that SPION-PEI nanoparticles loaded with siRNAs for Interleukin-2 and Interleukin-15 could be used to treat rheumatoid arthritis [54]. Stimulation with a neodymium magnet for two hours caused a significant increase in particle accumulation at the target joint, increased particle uptake into macrophages and T lymphocytes, and reduced cartilage destruction compared to unstimulated controls.

Furthermore, magnetic stimulation can be used to maintain drug elution rates as the carrier nears depletion. Mohapatra et al. demonstrated that brief magnetic stimulation of SPION-loaded chitosan-polyethylene glycol dimethacrylate microbeads could increase vancomycin elution to therapeutic levels (>2 mcg/mL) after drug release had dropped to negligible levels for three days [55,56]. This approach may be valuable as a means to reduce the dosing frequency.

### 3.4. Electric-Responsiveness

Electric stimuli-responsive systems are composed of electroactive polymers (EAPs), including polyaniline, polypyrrole, polythiophene, ethylene vinyl acetate, and polyethylene, that change shape, or volume upon stimulation with an electric current or a combination of both [57]. Alternating single and double bonds in the backbone of EAPs create a delocalized source of pi-bond electrons that can easily travel along the polymer chain, enabling the conduction of an electric charge [58]. Upon application of an electrical potential, EAPs will undergo reversible oxidation/reduction reactions that alter polymer charge, induce conformational changes, or both. The redox reactions are typically reversible, and many electro-responsive systems respond to repeat stimulation in a pulsatile on-off switch [59]. Ionic dopants are added during the polymerization process to control the initial redox state of the polymer and serve as ion carriers as the polymer charge changes during stimulation. Oxidation or reduction of the polymer may directly repel the drug payload, as with anionic ibuprofen, thereby increasing elution in response to stimulation [60,61]. Alternatively, the drug may bind to dopant molecules that carry it out of the polymer matrix upon stimulation, or the polymer undergoes conformation changes that allow increased diffusion [62]. 

EAPs typically suffer from inferior mechanical properties and are not biodegradable; therefore, many recent studies have incorporated EAPs into natural hydrogels or scaffolds to impart electro-responsive properties. For example, Atoufi et al. grafted aniline tetramers to alginate and combined the resulting EAP into agarose gels [59]. The resulting hydrogel had mechanical and biocompatibility properties comparable to typical agarose hydrogels but reproducibly released approximately 1.2% of the overall dexamethasone payload in response to three-minute stimulations with −1 V. Nano-scale conductive polymers have received increased interest due to their high drug binding efficiency and improved responsiveness to stimulation. Lee et al. created polypyrrole nanowire arrays from sacrificial alumina oxide templates. The resulting collection was found to adsorb 10× more DOX as bulk polypyrrole due to the increased surface area to volume ratio and exhibited pulsatile drug release when stimulated with −1 V [62]. Additionally, Wang et al. have used redox-induced EAP conformation changes to create functionalized β-cyclodextrin electro-responsive gates for mesoporous silica nanoparticles [63]. The restraining of gemcitabine release to periods of stimulation with −1.5 V, demonstrated active holding of the gates in the open or closed position, achieving true on-off elution.

In addition to directly increasing drug release from conductive systems, electric stimulation can be used to enhance cellular uptake of drugs or nanoparticles in a process known as electroporation. Application of DC or AC electrical pulses can create transient pores (lasting <15 min) in the cellular membranes, blood vessels, and skin, facilitating the delivery of drugs, nanoparticles, or both [64,65]. These membrane pores allow drugs or nanoparticles to bypass acidic endosomal compartments altogether, improving the efficacy of drug delivery to the cytosol and alleviating requirements of the carrier to withstand/escape the acidic endosomal chamber [66]. By optimizing the properties of the electric stimulus, it is possible to create pores in the cellular membrane and liposomal drug carriers simultaneously, thereby using one stimulus to facilitate cellular entry and release the drug payload [67].

Electric stimulation provides a simple and inexpensive method of modulating drug release as clinicians deem necessary. EAPs can be used by themselves or incorporated into biocompatible polymers to create novel stimuli-responsive, biodegradable systems. The redox reactions driving stimuli responsiveness are reversible and reproducible, enabling reliable control over drug release from the system. The primary disadvantage of electric stimulation is the need to place electrodes in the polymer matrix, limiting use to topical or subdermal implants. However, the lack of specialized equipment and ease of use make electric stimulation well suited for use in these areas.

## 4. Biological Stimuli-Responsive Systems

### Enzyme-Responsiveness

**Figure 5:**
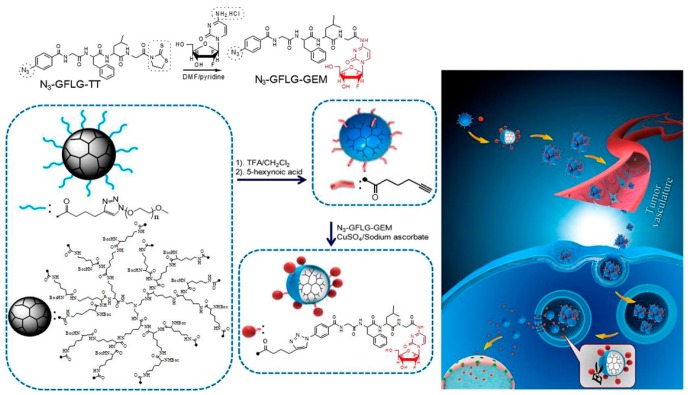
The scheme of preparing of lysine peptide dendrimer-glycly phenylalanyl leucyl glycine tetra-peptide-gemcitabine conjugate (Dendrimer-gemcitabine). The conjugate-based nanoparticles accumulate into the tumor via the EPR effect and enzyme-responsively release drugs. Reprinted with permission from *Acta Biomaterialia,* 2017, *55,* 153–162. Copyright (2017) Elsevier Ltd.

Due to their outstanding ability to bio-recognize and catalyze physicochemical material changes, enzymes are useful in the design of smart biomaterials. Enzyme-based smart delivery systems are optimal in certain applications when there is an overexpression of specific enzymes in the tissue environment, a concentration gradient difference related to a diseased condition, or the combination. Some cancerous tissues have been shown to possess elevated levels of matrix metalloproteinases (MMPs) [68], which possess the ability to selectively cleave peptide bonds between nonterminal amino acids [69]. Enzyme-responsive materials possess advantages due to their specificities in cell regulation and activities related to the variety of their biological and metabolic roles [70,71,72,73]. Enzymes secreted with spatiotemporal control possess some structural features that increase substrate specificity [74]. Enzyme-responsive systems can protect their cargo from degradation during transport to the target and release with selectivity at the target site (Figure 5) [75]. Enzymatic substrates covalently linked to amphiphilic copolymers is a common strategy for fabricating enzyme-responsive polymer assemblies [76,77]. Nanomaterials made from enzyme-responsive materials have the added advantage of increased permeability and retention (EPR) effects and site-specific delivery [78,79].

The amount of DOX released from an enzyme-responsive pillararene-based polymer substituted macrocyclic amphiphile system significantly increased in the presence of L-asparaginase [80]. Another group investigated a pillararene-based polymer-substituted macrocyclic amphiphile PPMA to release DOX in response to L-asparaginase [80]. Nanoparticles composed of poly(D, L-lactic-co-glycolic acid)-block-polyethylene glycol copolymer, blended with a tumor-activated prodrug, composed of an MMP2-sensitive peptide conjugating DOX to PLGA, released higher amounts of DOX when incubated with MMP2 [81]. The targeting of enzymatic substrates allows for improved specificity while mitigating undesired off-target effects. Wang et al. worked to combine montmorillonite (MMT) and hyaluronic acid (HA) on the surface of biomaterials within a multilayer film for long-term biofilm inhibition [82]. The films released higher levels of gentamicin sulfate in the presence of HAS compared to PBS, with release levels being directly correspondent to the HAS concentration [82]. When exposed to *Escherichia coli* and *Staphylococcus aureus* infection microenvironments, the films’ responsiveness to *E. coli* was higher [82].

**Figure 6:**
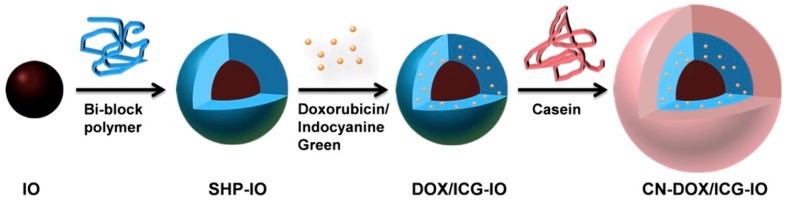
Illustration of layer-by-layer assembled casein coated iron oxide nanoparticles loaded with drug (DOX/Indocyanine green). Reprinted with permission from *Biomaterials,* 2015, *39,* 105–113. Copyright (2015) Elsevier Ltd.

Zhang et al. built an enzyme-responsive PEGylated system that was stimulated to release gemcitabine when exposed to Cathepsin B, which is expressed in the tumor microenvironment [75]. Cathepsin B presence or absence greatly influenced the amount of gemcitabine released [75]. Using layer-by-layer (LBL) composition, iron oxide nanoparticles coated with milk protein casein (CN) and loaded with DOX were investigated for oral delivery (Figure 6) [83]. Huang et al. found that DOX release from the nanoparticles increased as their exposure changed from simulated gastric to simulated intestinal juice, supporting their protective and enzyme-responsive properties [83]. Van Hove et al. developed an enzymatically-responsive PEG hydrogel containing pro-angiogenic peptides that demonstrated significant release in the presence of MMP2 as either cancer treatment or to reduce inflammation [84]. Peptide cleaving occurred at desired sites with no non-specific degradation transpiring when released from hydrogels [84]. Lee et al. investigated how to stabilize insulin at elevated temperatures by developing a novel trehalose-based hydrogel with the glucose-triggered release of insulin [85]. Data showed that the glucose-responsive trehalose hydrogel is effective against heating stress, retaining more than 50% of the loaded cargo at elevated temperatures [85]. The trehalose glycopolymers are active stabilizers for proteins, including insulin [86,87,88,89,90].

Enzyme-responsive systems have multiple applications across a myriad of diseases or ailments. An advantage of enzyme-responsive systems is the selectivity and built-in internally-stimulated mechanism, taking advantage of the pathological or physiological microenvironment, thereby reducing the potential for toxicity in healthy cells and tissues. Disadvantages include the release of the drug before reaching the intended target. For this reason, enzyme-responsive biomaterials possess other stimuli-responsive properties, i.e., pH, to protect the cargo until reaching the destination. Additionally, exposure of the biomaterial to its enzyme trigger or a closely related enzyme could release the load prematurely.

## 5. Multi Stimuli-Responsive Systems

The integration of multiple stimuli offers the opportunity to increase the fine-tuning of responses for each stimulus with the possibility of regulating the release profile. Multi-responsive systems provide the ability to preserve the primary drug until the intended target is reached. Recent studies have explored carriers to enhance drug delivery and simultaneously offer additional modes of treatment. Multi-response systems are addressing previous issues, such as low drug loading capacities, undesired drug release while in circulation, and any potential non-biodegradable properties, among many. Improving the goals of noninvasiveness and pinpointing intracellular release are some benefits of recent investigations. There remains a plethora of work to be done when it comes to meeting the challenges that any stimuli-responsive system faces but combining as many stimuli as needed offers solutions that were once not seen as possible.

Gold nanocages are investigated for therapeutic applications due to their porous walls, hollow interiors, and tunable localized plasma resonance peaks (LSPRs) that reside in the NIR region [91,92]. Wang et al. designed a multi-stimuli responsive nanosystem based on drug-loaded gold nanocages with hyaluronic acid with pinpointed intracellular drug release in conjunction with the synergistic combination of chemotherapy and phototherapy [73]. When no hyaluronidase was present, the release of encapsulated DOX was negligible in contrast to a significant burst release when exposed to hyaluronidase, with the addition of NIR irradiation increasing release [73]. A PEGylated multi-responsive copolymer-DOX prodrug model system was investigated for the delivery of hydrophobic drugs [93]. The presence or absence of GSH and Cathepsin B does not influence the release amount of DOX in more acidic conditions but increases the release rate when present, whereas at physiological pH, the enzymes have no effect [93]. To deliver drugs to breast and cervical cancer, Kashyap et al. designed a thermal and enzymatically responsive amphiphilic copolymer to release DOX, finding that polymers released 90% of the loaded DOX when temperatures were similar to cancerous tissues compared to only 20% at average physiological temperatures [94]. Similarly, these DOX-loaded polymers released 90% of the loaded drug within 12 h in the presence of esterase, and 20% in the absence [94].

Hervault et al. investigated a controlled drug delivery system with pH- and thermo-responsiveness for multi-modal cancer therapy through the combination of magnetic targeting and hyperthermia, through the formation of pH-sensitive imine bonds between the amine group of DOX and the polymer’s aldehyde group [95]. The maximal release of DOX occurred at a pH of 5.7 and 50 °C when exposed to both stimuli, concurrently [95]. Li et al. composed dual-responsive hybrid mats with ketoprofen (KET), poly(N-vinylcaprolactam), ethyl cellulose, and Eudragit L100 in various combinations as a drug delivery system that demonstrated a pH-dependence independent of temperature, with higher responses in less acidic environments [96]. KET released from the mats when exposed to dual stimuli emulated the results achieved when singularly stimulated, with the most significant and rapid release occurring at pH 7.4 and 25 °C [96].

Daravan et al. investigated a thermal- and pH-responsive ABC triblock copolymer for enhancing the delivery of DOX, which demonstrated a faster release in acidic conditions, with release decreasing as a function of increasing pH at the average body temperature [97]. When the temperature rose to 46 °C, the release rates for all pH values decreased [97]. For on-demand activation after light excitation, de Solorzano et al. synthesized a novel amine-terminated P (MEO_2_MA-co-OEGMA) surface-grafted to plasmonic copper sulfide nanoparticles, which produced a cleavable thermo-responsive nanocomposite, to host and release bupivacaine anesthetic [98]. As the temperature increased from 37 to 45 °C, drug release occurred at higher rates, with even higher release achieved when composites were stepwise irradiated with an 808 nm laser (1.89 W cm^−2^) [98]. Photothermal-chemotherapy (PT-CT) has promise, but due to issues related to the safety of carriers and drug release profiles, this led to a system consisting of PEG-modified polydopamine nanoparticles (PDA-PEG) loaded with DOX to treat cancer with NIR-, pH-, and redox-stimuli capability [99]. DOX release from PDA-PEG, when exposed to NIR irradiation, increased as a function of time and in acidic conditions [99].

Multi-responsive systems allow researchers to fine-tune systems to meet current challenges that may have been more difficult to overcome. Protection of the cargo is a primary advantage that multi-responsive systems possess over their mono-responsive counterparts. Recent investigations have increased modes of layered stimuli to at times three or more, offering higher chances for success. The complexity of these systems and their fabrication can be a limiting factor as investigators seek clinical translation.

## 6. Conclusions

Stimuli-responsive systems have evolved to respond to various modes of internal- and external-stimuli, or both. These advances present researchers and clinicians with great promise in enhancing current treatment options as well as future solutions. Over the past decade, stimuli-responsive systems have expanded the horizon of smart biomaterials while improving precision to improve the efficacy and reduce off-target toxicity of therapeutic molecules. Chemical, physical, and biological stimuli may be internal or externally triggered to boost or trigger drug release in particular tissue or disease states. While several recent works have advanced the field, primary limitations for each of these types of smart polymer biomaterials rests on the ability to penetrate deeper layers of tissue and limiting unintended tissue damage for physical-based triggers. Ongoing efforts to keep the payload retained until desired stimulation is a recurring limiting factor that requires future progress. While results in vitro and from preclinical studies provide promising evidence, few polymeric smart biomaterials have advanced to clinical studies of efficacy and safety. Clinical evaluations of safety are needed to improve the future of smart drug delivery vehicles.

## Figures and Tables

**Table 1:** Summary of featured stimuli-responsive polymers.

Stimuli	Polymer	Major Result(s)	Ref(s)
pH	N-carboxyethyl chitosan/dibezaldehyde-terminated poly(ethylene glycol)	pH changes promote chemical and physical modifications that swell the system inducing cargo release	Qu et al. [6]
pH	Poly(lactic acid)-poly(ethyleneimine)	Burst release of doxorubicin (DOX) as pH shifted from 7.4 to 5.4	Li et al. [2]
pH	Poly(lactic-co-glycolic acid) (PLGA)	Morphological change induces drug release	Chung et al. [11]
pH	Poly(acrylamide)	Drug release at pH > 4.0	Pafiti et al. [18]
Ultrasound	Poly(ethylene glycol)	Led to a six-fold increase in the cumulative release	Kearney et al. [19]
Ultrasound	Alginate	Pulsed stimulation outperformed constant stimulation	Huebsch et al. [20]
Ultrasound	Chitosan	Significant release compared to no stimulus	Zhou et al. [21]
Ultrasound	Poly(methacrylic acid) (PMAA)	Design a three in one theranostic nanoplatform for imaging and release	Yang et al. [22]
Ultrasound	Poly(2-oxazoline) micelles	Possible carrier with increased release	Salgarella et al. [25]
Ultrasound	polylactic acid (PLA)	Long-term encapsulation of small hydrophilic molecules and four times the release profile with HIFU	Gai et al. [26]
UV	Spiropyran-hyperbranched polyglycerol micelle	Assembly and disassembly of micelle induced by UV light exposure controls the drug release. Superior biocompatibility with cells in the absence of UV	Son et al. [34]
UV	Azobenzene-β-galactose micelle	Short UV exposure (2 min) to release drug; low cytotoxicity of unloaded micelles	Pearson et al. [36]
UV	2-hydroxyethyl methacrylate and ethylene glycol dimethacrylate	Deliver multiple doses of drug upon UV exposure over a prolonged period of time (≤160 h)	Hardy et al. [37]
UV	mPEG-PLGA nanoparticle	Reverse multidrug resistance of tumor cells; enhance chemosensitization of cells to DOX therapy	Fan et al. [38]
NIR	Diselenide-cross-linked poly(methacrylic acid)	Controlled illumination with specific number of irradiation times allowed for on-demand controlled drug release and nanogel degradation. Rapid internalization by HeLa cell and cytotoxic under NIR irradiation	Tian et al. [40]
NIR	Β-cylcodextrin	Anticancer activity in vitro and in vivo against breast cancer, with accelerated drug release upon NIR exposure	Liang et al. [41]
NIR	Polycaprolactone	On-demand, stepwise drug-release after multiple cycles of NIR exposure with low off-state leakage.	Chen et al. [42]
Red light	Tetra-ortho-methoxy-substituted azobenzene & β-cyclodextrin	Responsive to red light instead of UV. Deeper tissue penetration depth	Wang et al. [44]
AMF	Aminosilan-type shell	EMF stimulation of SPIONS can maintain elevated temperatures of approximately 45 °C in glioblastoma multiforme tumors	Maier-Hauff et al. [47]
AMF	Polyethylene glycol w/azo drug linker	SPION local temperature can increase up to 50 °C without inducing significant temperature increases in media at sufficiently low concentrations	Riedinger et al. [48]
AMF	(N-isopropylacrylamide)-(N-hydroxymethyl) acrylamide	SPION stimulation can trigger PNIPAM critical temperature transition without increasing temperature of surrounding media	Guisasola et al. [49]
AMF	Poly(maleic anhydride-alt-1-octadecene)	Distance from the nanoparticle surface can be used to control temperature dependent effects during AMF stimulation	Dias et al. [50]
AMF	PLGA	SPION stimulation induced drug release by increasing temperature above the glass transition of PLGA	Thirunavukkarasu et al. [51]
Permanent magnet	Tetramethylazanium hydroxide	Intrathecally delivered SPIONS loaded with NSAIDS produced magnetic field dependent reductions in pain and inflammatory markers in a murine model	Wu et al. [52]
Permanent magnet	Polyethyleneimine	External magnetic guidance improved accumulation of SPIONS in arthritic joints in a rat model	Duan et al. [54]
AMF	Chitosan-polyethylene glycol	SPION loaded microbeads can respond to multiple stimuli and increase drug release to efficacious levels as the carrier nears exhaustion	Mohapatra et al. [56]
Electric	Agarose/alginate-aniline tetramer	Conductive tetramers improve hydrogel biocompatability with neural cells and enables repeat stimuli responsive drug release	Atoufi et al. [59]
Electric	Poly(3,4-ethylenedioxypyrrole)	Stimulation induces rapid release of ionically bound ibuprofen but not ibuprofen physically entrapped in the matrix during electrochemical polymerization	Krukiewicz et al. [60]
Electric	Poly(3-methoxydiphenylamine)/Pectin blend	Stimulation increased hydrogel mesh pore size allowing increased drug elution	Mongkolkitikul et al. [61]
Electric	Polypyrrole	Sacrificial templates can be used to create electrically responsive nanowires	Lee et al. [62]
Electric	Monoferrocene functionalized β-cyclodextrin	Stimulus-induced conformational changes can be used to control polymeric ‘gates’ for on/off delivery using mesoporous particles	Wang et al. [63]
Enzyme	PEGylated alkynylated peptide dendrimer	Minimal release in the absence of Cathepsin B	Zhang et al. [75]
Enzyme	Polydimethylsiloxane, polyethylenimine	Release in the presence of HAS, *E. coli*, or *S. aureus*	Wang et al. [82]
Enzyme	Poly(maleic acid)	No release until exposure to intestine protease trypsin	Huang et al. [83]
Enzyme	Poly(ethylene glycol)	Peptide cleaving at desired sites	Van Hove et al. [84]
Enzyme	Poly(styrenyl ether trehalose), poly(ethylene glycol)	Ability to withstand elevated temperatures with cargo intact	Lee et al. [85]
Enzyme, NIR	Poly(vinyl pyrrolidone)	Minimal release in the absence of hyaluronidase, NIR promoting more release	Wang et al. [73]
Enzyme, pH	Poly(ethylene glycol)	Release rate increase at pH 5.4 in presence of cathepsin B and glutathione	Duan et al. [93]
Enzyme, Thermal	3-pentadecylphenol, oligoethylene glycol acrylate	Proposed release at tissue based on temperature with intracellular release concurrent with enzyme exposure	Kashyap et al. [94]
pH, Thermal	Poly(ethylene glycol) methyl ether methacrylate	pH and temperature greatly influence the release of DOX	Hervault et al. [95]
pH, Thermal	Poly(N-vinylcaprolactam), ethyl cellulose, Eudagrit L100	Most pronounced release occurred at 25 °C and pH 7.4	Li et al. [96]
pH, Thermal	Poly(2-succinyloxyethyl methacrylate)-*b*-(*N*-isopropylacrylamide)-*b*-[(*N*-4-vinylbenzyl),*N*,*N*-diethylamine]], [P(SEMA-*b*-NIPAAm-*b*-VEA)]	Greatest DOX release observed at 37 °C and pH 4, increase in temperature led to decrease in DOX release	Davaran et al. [97]
NIR, Thermal	Poly(ethylene glycol) methyl ether methacrylate, poly(vinyl pyrrolidone)	Release was higher at 45 °C with a burst increase synonymous with NIR irradiation	Ortiz de Solorzano et al. [98]
NIR, pH, Redox	Poly(ethylene glycol), poly(dopamine)	NIR irradiation release is function of exposure time, pH and redox release greatest at pH 7.4	Wang et al. [99]
